# In vivo T_1_ mapping of neonatal brain tissue at 64 mT

**DOI:** 10.1002/mrm.29509

**Published:** 2022-11-13

**Authors:** Francesco Padormo, Paul Cawley, Louise Dillon, Emer Hughes, Jennifer Almalbis, Joanna Robinson, Alessandra Maggioni, Miguel De La Fuente Botella, Dan Cromb, Anthony Price, Lori Arlinghaus, John Pitts, Tianrui Luo, Dingtian Zhang, Sean C. L. Deoni, Steve Williams, Shaihan Malik, Jonathan O′Muircheartaigh, Serena J. Counsell, Mary Rutherford, Tomoki Arichi, A. David Edwards, Joseph V. Hajnal

**Affiliations:** ^1^ Center for the Developing Brain, School of Imaging Sciences and Biomedical Engineering King′s College London London United Kingdom; ^2^ Medical Physics Guy′s & St. Thomas' NHS Foundation Trust London United Kingdom; ^3^ Hyperfine, Inc. Guilford Connecticut USA; ^4^ Medical Research Council Center for Neurodevelopmental Disorders King′s College London London United Kingdom; ^5^ Department of Neonatology Guy′s and St. Thomas′ NHS Foundation Trust London United Kingdom; ^6^ Advanced Baby Imaging Lab, Rhode Island Hospital Warren, Alpert Medical School at Brown University Providence Rhode Island USA; ^7^ Department of Diagnostic Radiology Warren Alpert Medical School at Brown University Providence Rhode Island USA; ^8^ Department of Pediatrics Warren Alpert Medical School at Brown University Providence Rhode Island USA; ^9^ Center for Neuroimaging Sciences King′s College London London United Kingdom; ^10^ Department of Forensic and Neurodevelopmental Science, Institute of Psychiatry, Psychology and Neuroscience King′s College London London United Kingdom; ^11^ Department of Bioengineering Imperial College London London United Kingdom; ^12^ Pediatric Neurosciences, Evelina London Children′s Hospital Guys′ and St. Thomas′ NHS Foundation Trust London United Kingdom

**Keywords:** gray matter, neonatal, relaxometry, ultralow‐field MRI, white matter

## Abstract

**Purpose:**

Ultralow‐field (ULF) point‐of‐care MRI systems allow image acquisition without interrupting medical provision, with neonatal clinical care being an important potential application. The ability to measure neonatal brain tissue T_1_ is a key enabling technology for subsequent structural image contrast optimization, as well as being a potential biomarker for brain development. Here we describe an optimized strategy for neonatal T_1_ mapping at ULF.

**Methods:**

Examinations were performed on a 64‐mT portable MRI system. A phantom validation experiment was performed, and a total of 33 in vivo exams were acquired from 28 neonates with postmenstrual age ranging from 31^+4^ to 49^+0^ weeks. Multiple inversion‐recovery turbo spin‐echo sequences were acquired with differing inversion and repetition times. An analysis pipeline incorporating inter‐sequence motion correction generated proton density and T_1_ maps. Regions of interest were placed in the cerebral deep gray matter, frontal white matter, and cerebellum. Weighted linear regression was used to predict T_1_ as a function of postmenstrual age.

**Results:**

Reduction of T_1_ with postmenstrual age is observed in all measured brain tissue; the change in T_1_ per week and 95% confidence intervals is given by dT_1_ = −21 ms/week [−25, −16] (cerebellum), dT_1_ = −14 ms/week [−18, −10] (deep gray matter), and dT_1_ = −35 ms/week [−45, −25] (white matter).

**Conclusion:**

Neonatal T_1_ values at ULF are shorter than those previously described at standard clinical field strengths, but longer than those of adults at ULF. T_1_ reduces with postmenstrual age and is therefore a candidate biomarker for perinatal brain development.

## 1 INTRODUCTION

Recent years have seen increased popularity of MRI systems using magnetic field strengths far below those of traditional clinical systems with superconducting magnets. These systems have been engineered to optimize alternative design criteria (such as increased portability and lowered cost) in contrast to high‐field systems, which generally aim to maximize image quality. Crucially, these low‐field systems can also have reduced infrastructure needs, promising to expand the use of MRI beyond radiology departments in high‐income countries.

Ultralow‐field (ULF) point‐of‐care MRI systems^1^, ^2^, ^3^, ^4^, ^5^ are a category of device designed to allow image acquisition without interrupting a patient′s medical provision. Neonatal clinical care is an important potential application, as portable systems would allow imaging on neonatal intensive care units in both high and lower‐resourced settings and where rapid diagnostic information could have large implications for clinical decision making. Key examples include neonates suffering from neurological pathologies such as hypoxic ischemic injury, hydrocephalus, stroke, as well as other conditions elsewhere in the body.

Although neonatal brain MRI has been performed at a variety of field strengths,^6^, ^7^, ^8^, ^9^, ^10^ there is limited work in this cohort at ULF. While ULF adult imaging has been successfully demonstrated,^11^ initial local experience demonstrates that sequences and acquisition parameters optimized for adults do not necessarily provide optimal image contrast in younger cohorts. This phenomenon is likely due to marked relaxation rate differences between adult and neonatal brain tissues, demonstrated at both high‐field in vivo^12^, ^13^, ^14^, ^15^, ^16^, ^17^ and at ULF in ex vivo tissue samples.^18^

The ability to measure neonatal brain tissue T_1_ is a key enabling technology for further ULF neonatal imaging. It will allow image‐contrast optimization for structural imaging, as well as having the potential to be a biomarker for neonatal brain development. However, there has been no in vivo ULF neonatal T_1_ mapping to date, motivating this work using a 64‐mT portable MRI system. Here we describe an optimized strategy for T_1_ mapping using an inversion‐recovery turbo spin‐echo sequence and report values in 28 neonates.

## 2 METHODS

We designed a combined acquisition and analysis strategy to allow T_1_ mapping in the presence of participant motion, typical in neonates and infants. Multiple T_1_‐weighted volumes are first acquired, with each acquisition designed to have short duration to minimize the chance of intravolume motion corruption.

The reconstruction pipeline then takes an iterative two‐step approach. First, data fitting is performed without any correction for intersequence motion. The resulting proton density (M_0_) and T_1_ maps are then used to synthesize new images with the same T_1_ weighting as the acquired data, to which the original images are registered. These registered images are then used for data fitting, with this process repeated for a fixed number of iterations.

### 2.1 Acquisition

Two phantom experiments were performed to validate the acquisition and analysis pipeline used for our in vivo neonatal studies. A contrast phantom consisted of six 50‐ml falcon tubes containing distilled water and MnCl_2_ at concentrations of 0, 0.01, 0.05, 0.1, 0.15, and 0.2 mM, placed in a custom‐built holder so that the long axes of the vials were aligned with the main magnetic field. The second, a homogeneity phantom, was a spherical flask of 144‐mm diameter containing a solution of saline and gadolinium doped to achieve a T_1_ in the range of neonatal white matter at 64 mT.

A total of 33 exams were acquired from 28 neonates (mean gestation at birth: 36^+3^ weeks^+days^, range: [27^+0^, 40^+4^]; mean age at first scan: 13 days, range: [1, 94]; mean postmenstrual age at scan: 39^+2^ weeks^+days^, range: [31^+4^, 49^+0^]) as part of two National Health Service UK Research Ethics Committee approved studies (12/LO/1247 and 19/LO/1384). Recruitment to these studies included healthy controls and clinically referred neonates. Infants referred for clinical scans were eligible for chloral hydrate sedation. All medical support requirements, such as ventilation, intravenous infusions and/or thermoregulation, were continued throughout scanning, alongside continuous oxygen saturation and heart‐rate monitoring. Subjects were swaddled and immobilized using a vacuum‐evacuated bag containing polystyrene beads and placed in a neonatal imaging cradle designed to position the neonate′s head at the magnet isocenter. Imaging was performed with a 64‐mT Swoop portable MRI system (Hyperfine, Guilford, CT), using the built‐in RF interference rejection method^19^ and single‐channel transmit/eight‐channel receive coil.

**FIGURE 1:**
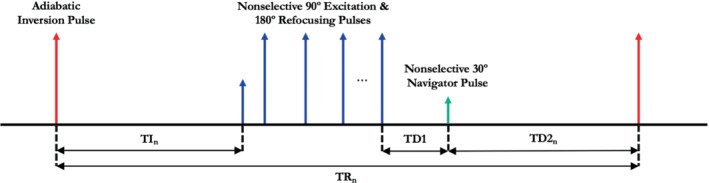
Pulse‐sequence diagram of inversion‐recovery 3D turbo spin echo. The sequence consists of an adiabatic inversion pulse, a turbo spin echo readout train, and a center‐frequency navigator

The scanning protocol consisted of a prescan calibration, localizer, and several acquisitions of the Hyperfine product inversion‐recovery 3D turbo spin‐echo acquisition (Figure 1), each modified using the sequence development interface to have differing TRs and TIs (TR_n_ and TI_n_, with the subscript indexing the multiple acquisitions). The base sequence contained an adiabatic inversion pulse, a T_1_ recovery period (TI_n_), a nonselective turbo spin‐echo readout of N_TF_ echoes using low‐high phase‐encode ordering, a delay period TD1 between the readout and a center‐frequency navigator echo generated by a nonselective excitation of flip angle $\theta_{\mathrm{nav}}$, followed by a recovery time TD2_n_. The signal S_n_ at the first echo for a given choice (TR_n_, TI_n_) is given by Equation 1; note that the contribution from nonzero TE is subsumed into the M_0_ term.

(1) $$\begin{array}{cc} S_{n}\left(M_{0},T_{1};{\mathrm{TR}}_{n},{\mathrm{TI}}_{n}\right) & =M_{0}\left(1-e^{-{\mathrm{TI}}_{n}/T_{1}}\right)\\ & \;-M_{0}\left(\left(1-e^{-\mathrm{TD}1/T_{1}}\right)\cos\left(\theta_{\mathrm{nav}}\right)e^{-\mathrm{TD}2_{n}/T_{1}}\right.\\ & \;\left.+\left(1-e^{-\mathrm{TD}2_{n}/T_{1}}\right)\right)e^{-{\mathrm{TI}}_{n}/T_{1}} \end{array}$$

The following base sequence parameters were used for all acquisitions: FOV = 180 × 220 × 180 mm (right–left × anterior–posterior × feet–head), resolution = 2.8 × 2.8 × 2.8 mm, turbo factor (N_TF_) = 48, echo spacing (τ) = 4.62 ms, bandwidth = 64 kHz, excitation flip angle = 90°, refocusing flip angle = 180°, TE = 4.62 ms, $\theta_{\mathrm{nav}}$ = 30°, TD1 = 239 ms, and TD2_n_ = TR_n_‐TI_n_‐N_TF_ τ‐TD1. Preliminary experiments confirmed insensitivity to transmitter voltage miscalibration.

The comprehensive mapping protocol is given by row 0 in Table 1. This was used exclusively for the phantom acquisitions, with the protocol acquired at lower resolution (5 × 5 × 5 mm) for the homogeneity phantom to increase measurement SNR. Additionally, the homogeneity phantom experiment included the acquisition of a B_1_^+^ map using actual flip‐angle imaging^20^ with the following sequence parameters: TR1 = 20 ms, TR2 = 100 ms, TE = 2.56 ms, flip angle = 90°, bandwidth = 20 kHz, FOV = 210 × 210 × 210 mm, and resolution = 5 × 5 × 5 mm.

**TABLE 1:** Imaging parameters of the protocols used within the presented study and number of acquisitions that each protocol used

Protocol #	Acquisition protocol	In vivo acquisitions (*N*)
0	TI = [100, 200, 300, 400, 500, 600, 700, 800, 1010, 900] TR = [1500, 1500, 1500, 1500, 1500, 1500, 1500, 1500, 1500, 3000]	0
1	TI = [100, 300, 400, 500, 600, 800, 1010, 900] TR = [1500, 1500, 1500, 1500, 1500, 1500, 1500, 3000]	12
2	TI = [100, 300, 500, 600, 800, 1010, 900] TR = [1500, 1500, 1500, 1500, 1500, 1500, 3000]	8
3	TI = [100, 300, 400, 500, 600, 800, 1010] TR = [1500, 1500, 1500, 1500, 1500, 1500, 1500]	1
4	TI = [400, 500, 600, 800, 1010, 900] TR = [1500, 1500, 1500, 1500, 1500, 3000]	1
5	TI = [100, 200, 300, 400, 500, 600, 700, 800, 1010] TR = [1500, 1500, 1500, 1500, 1500, 1500, 1500, 1500, 1500]	2
6	TI = [100, 200, 300, 400, 500, 600, 700, 800] TR = [1500, 1500, 1500, 1500, 1500, 1500, 1500, 1500]	4
7	TI = [100, 200, 300, 400, 500, 600] TR = [1500, 1500, 1500, 1500, 1500, 1500]	5

Other variants were also used (rows 1–7), reflecting the need to limit total acquisition time for neonatal scanning as per study protocol (protocol 1), abbreviated scanning sessions due to other clinical priorities and neonates awaking from sleep (protocols 2–4), or initial trial versions of the protocol before modification to improve performance for longer relaxation times (protocols 5–7). All data are included to maximize the number of subjects included in the study. Additionally, note that the listed inversion times are those entered on the system′s user interface; the physical inversion times are 5.75 ms longer. Sequences using TR = 1500 ms had a total duration of 3 min 32 s; sequences using TR = 3000 ms had a total duration of 7 min 4 s.

### 2.2 Reconstruction—data fitting

Magnitude images were exported from the scanner in DICOM format, with the following steps performed for each participant. An initial brain and scalp mask was generated using FSL BET^21^ with fractional threshold set to 0.1. This mask was manually edited to exclude the face, mouth, and all anatomy inferior to the brain to allow use of rigid‐body registration in subsequent processing steps. Phantom images were masked by image thresholding using *MATLAB* (The MathWorks, Natick, MA, USA).

Fitting was performed using *MATLAB*. The low SNR of ULF MRI and the magnitude‐only image intensities available through data export results in a Rician distribution of image noise.^22^ It is appropriate to use statistical parameter‐estimation methods designed for the statistical noise distribution present in the data. In this case, the optimization problem is obtained by maximizing the log‐likelihood function given Rician‐distributed noise,^23^ as follows:

(2) $$\begin{array}{cc} \min_{M_{0},T_{1}}\Phi = & \sum_{n=1}^{N}\frac{{\left|S_{n}\left({\mathrm{TR}}_{n},{\mathrm{TI}}_{n};M_{0},T_{1}\right)\right|}^{2}}{2\sigma^{2}}\\ & -\ln\left(I_{0}\left(\frac{d_{n}\left|S_{n}\left({\mathrm{TR}}_{n},{\mathrm{TI}}_{n};M_{0},T_{1}\right)\right|}{\sigma^{2}}\right)\right) \end{array}$$

where Φ is the cost function; *n* is an index over the *N* acquisitions; σ is the SD of the underlying Gaussian‐distributed noise; I_0_ is the zeroth‐order modified Bessel function; and d_n_ is the measured signal of the *n*th acquisition. An estimate of σ is obtained by finding the mean signal ${\bar{\sigma }}_{\text{Rician}}$ in a region of interest (ROI), manually placed in a region avoiding signal and artifacts, and using the relation $\sigma ={\bar{\sigma }}_{\text{Rician}}/\sqrt{\pi /2}$. The cost function was minimized for each voxel in the mask using a two‐step procedure. A coarse search was first performed (160000 M_0_ and T_1_ combinations, range: [0, 1000] and [50 ms, 4000 ms], respectively) to obtain an estimate of the solution. This was used as the initial point when minimizing the cost function using the fmincon routine, with the solutions constrained to lie in the ranges used in the coarse search. This process yielded M_0_ and T_1_ maps.

### 2.3 Reconstruction—motion compensation

These initial M_0_ and T_1_ maps were then used in conjunction with Equation 1 to generate synthetic images for each acquired sequence. These synthetic images were used as registration targets to which the acquired images were registered. The registration is performed with FSL FLIRT^24^, ^25^ using a rigid‐body transformation and sinc interpolation, with the previously defined mask used to exclude areas that may have undergone nonrigid motion. These registered acquired images were then again used for data fitting. The whole process was repeated until convergence, with local experience indicating that three iterations were sufficient.

### 2.4 Phantom data analysis

Both phantom data sets (acquired as per protocol 0 in Table 1) were reconstructed with the pipeline outlined previously. However, the contrast phantom data were reconstructed eight times in total, each using the appropriate set of source images as prescribed by protocols 0–7 in Table 1. The T_1_ of all voxels within each vial were extracted, and the mean and SD calculated. To assess the validity of the acquisition, each protocol and the reconstruction pipeline, a linear weighted least‐squares fit was performed for each protocol and results plotted against concentration (c) of MnCl_2_ to confirm consistency with the known relationship given by Equation 3, where T_1_(c = 0) is the unknown relaxation time of the distilled water at 64 mT, and *R* is the concentration of added salt (in mM). The *r* ^2^ for each fit was used to evaluate data to model consistency.

(3) $$1/T_{1}\left(c\right)=1/T_{1}\left(c=0\right)+\mathrm{Rc}$$

### 2.5 In vivo ROI analysis

The final set of synthesized in vivo neonatal images were visually examined by a single reader (FP) to place ROIs in the deep gray matter (putamen/caudate), frontal white matter, and cerebellum. For each anatomical location, ROIs of size 2 × 2 × 2 voxels were placed on both the left and right of the brain. The mean and SD T_1_ values were calculated for each anatomical location to obtain per subject values. A group analysis was performed by calculating a weighted linear regression to predict T_1_ as a function of postmenstrual age; the weights were taken as the inverse square of the SDs.

## 3 RESULTS

**FIGURE 2:**
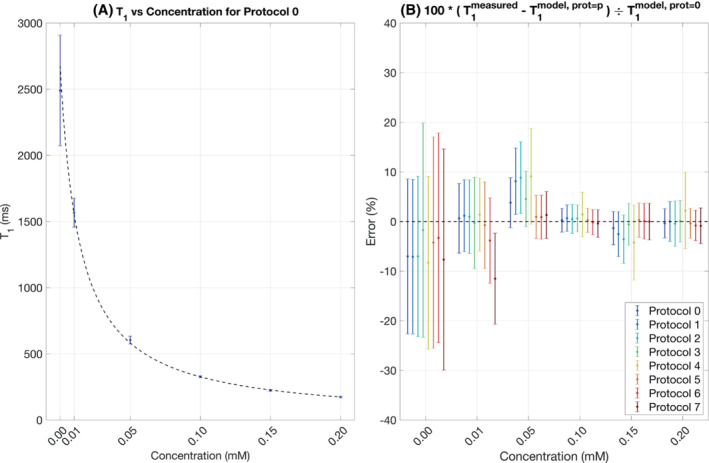
Results of the contrast phantom validation experiment.

Figure 2 shows the results of the contrast phantom validation experiment. Figure 2A shows the measured T_1_ versus concentration for protocol 0, with datapoints and error bars indicating the mean and SDs of all voxels within each vial. The dashed black lines indicate the fit to Equation 3. The *r* ^2^ values of the fits to Equation 3 were greater than 0.997 for all protocols, indicating that the pulse sequence, protocols, and reconstruction pipeline are valid.

Figure 2B shows the results of reconstructing the contrast phantom data presuming the acquisition protocols outlined in Table 1. The vertical axes show the fractional difference between the measured T_1_s versus those predicted via the model fit for protocol 0. All but three measurements (protocol 7, 0.01 mM; protocols 1 and 2, 0.05 mM) are within 1 SD of the model. Different protocols show different degrees of bias and variance as compared with protocol 0, and differences are seen as a function of T_1_.

**FIGURE 3:**
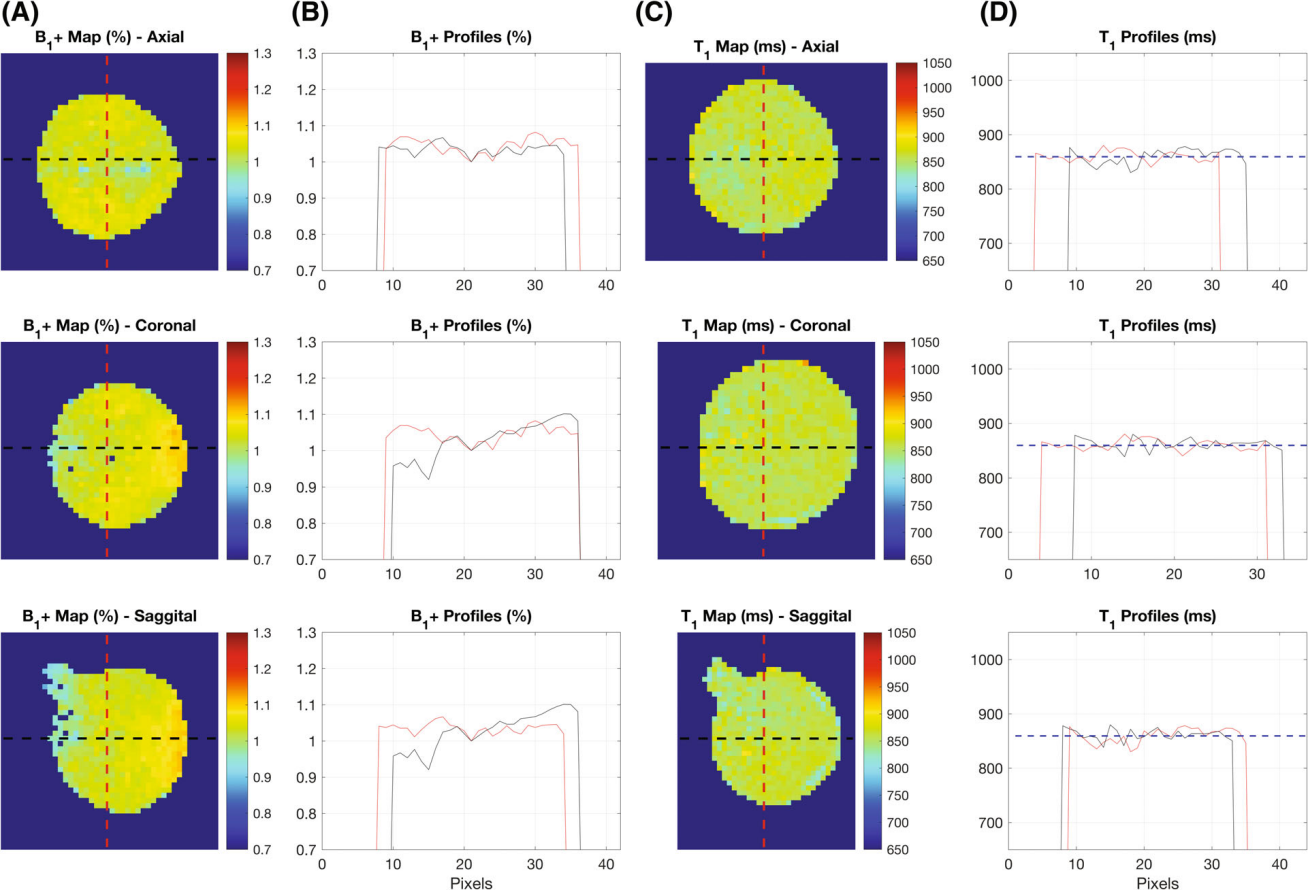
Results of the homogeneity phantom validation experiment

The results of the homogeneity phantom experiment are shown in Figure 3. Figure 3A shows the B_1_^+^ of three orthogonal slices through the phantom. Although there is little appreciable inhomogeneity in the transverse plane (top row), there is a 15%–20% variation in the superior–inferior direction. This is shown further by the black line profiles in the second and third rows of Figure 3B. Despite the B_1_^+^ inhomogeneity present, Figure 3C,D demonstrates that this does not introduce spatial variation in the T_1_ measurements.

Supporting Information Videos S1 and S2 show the effect of the intersequence motion‐correction pipeline for 3 participants with varying degrees of motion. In each case, the top row shows the source data before intersequence motion correction, and the bottom row shows the source data after three iterations of the proposed reconstruction pipeline. Although there is still motion present in the images after correction, motion is only present outside of the brain parenchyma; the brain after correction is static.

**FIGURE 4:**
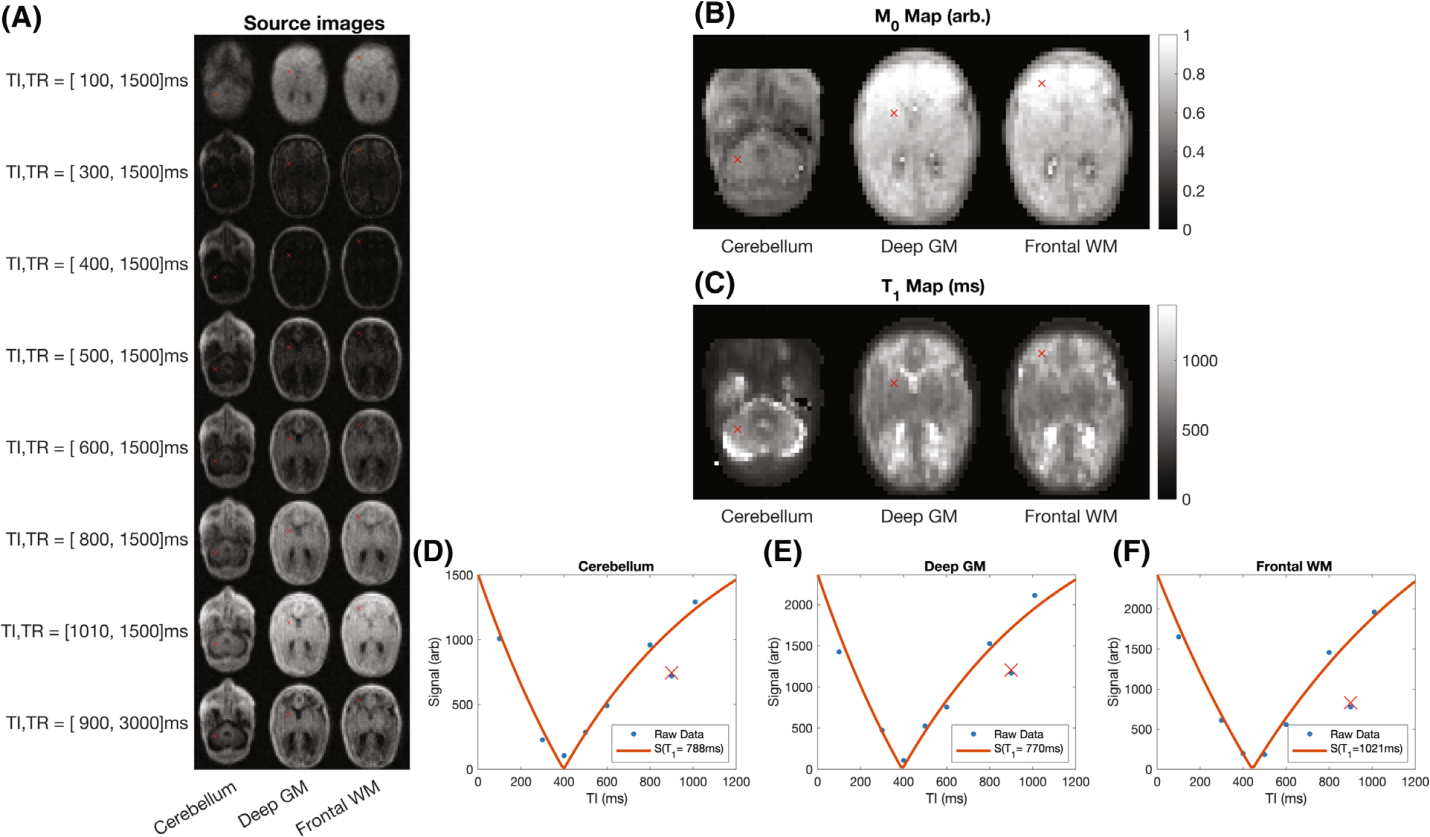
Three slices from a single neonate (gestation 33^+2^, postmenstrual age 34^+0^). A, Source images for each T_1_‐weighted acquisition. B,C, M_0_ and T_1_ maps. D–F, Raw datapoints (blue dots) and predicted signals (red lines/cross) based on measured T_1_ and M_0_. The blue datapoint/red cross away from the curve corresponds to the TR = 3000 ms datapoint, and therefore belongs to a separate inversion‐recovery curve.

Example data from 1 subject (gestation 33^+2^, postmenstrual age 34^+0^) are shown in Figure 4. Figure 4A shows the three axial slices containing the selected ROIs for all acquired sequences, with the center of each ROI in a single hemisphere marked. Figure 4B,C shows M_0_ and T_1_ maps for the same slices. Figure 4D–F shows the intensity of the center pixel of each selected ROI (blue points). To allow visual evaluation of the fitting procedure, the measured M_0_ and T_1_ were used in conjunction with Equation 1 to predict the signal as a function of TI assuming TR = 1500 ms (red line) and for TI = 900 ms and TR = 3000 ms (red cross).

**FIGURE 5:**
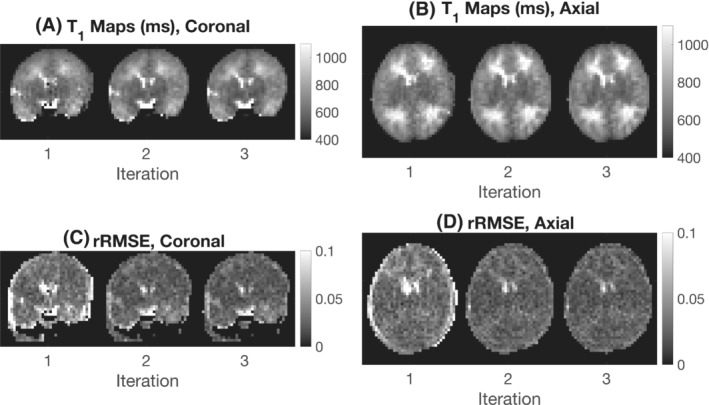
The effect of motion correction on T_1_ maps of a neonate born at 38^+3^ gestation and imaged at 41^+0^ postmenstrual age. A,B, Coronal and axial T_1_ maps for three iterations of the reconstruction pipeline. C,D, Coronal and axial relative RMS error maps between the input images and predicted output images at each iteration

The impact of image registration on T_1_ map quality is shown in Figure 5. Figure 5A,B shows coronal and axial T_1_ maps for the three iterations of the reconstruction pipeline. Later iterations of the T_1_ maps have fewer spurious individual pixels intensities, as for example in the deep gray matter that has a more uniform appearance. Figure 5C,D shows RMS error maps (between the input images and the predicted images) as a fraction of the average input image pixel intensity across acquisitions at each iteration. The relative RMS error decreases for increasing number of iterations.

**FIGURE 6:**
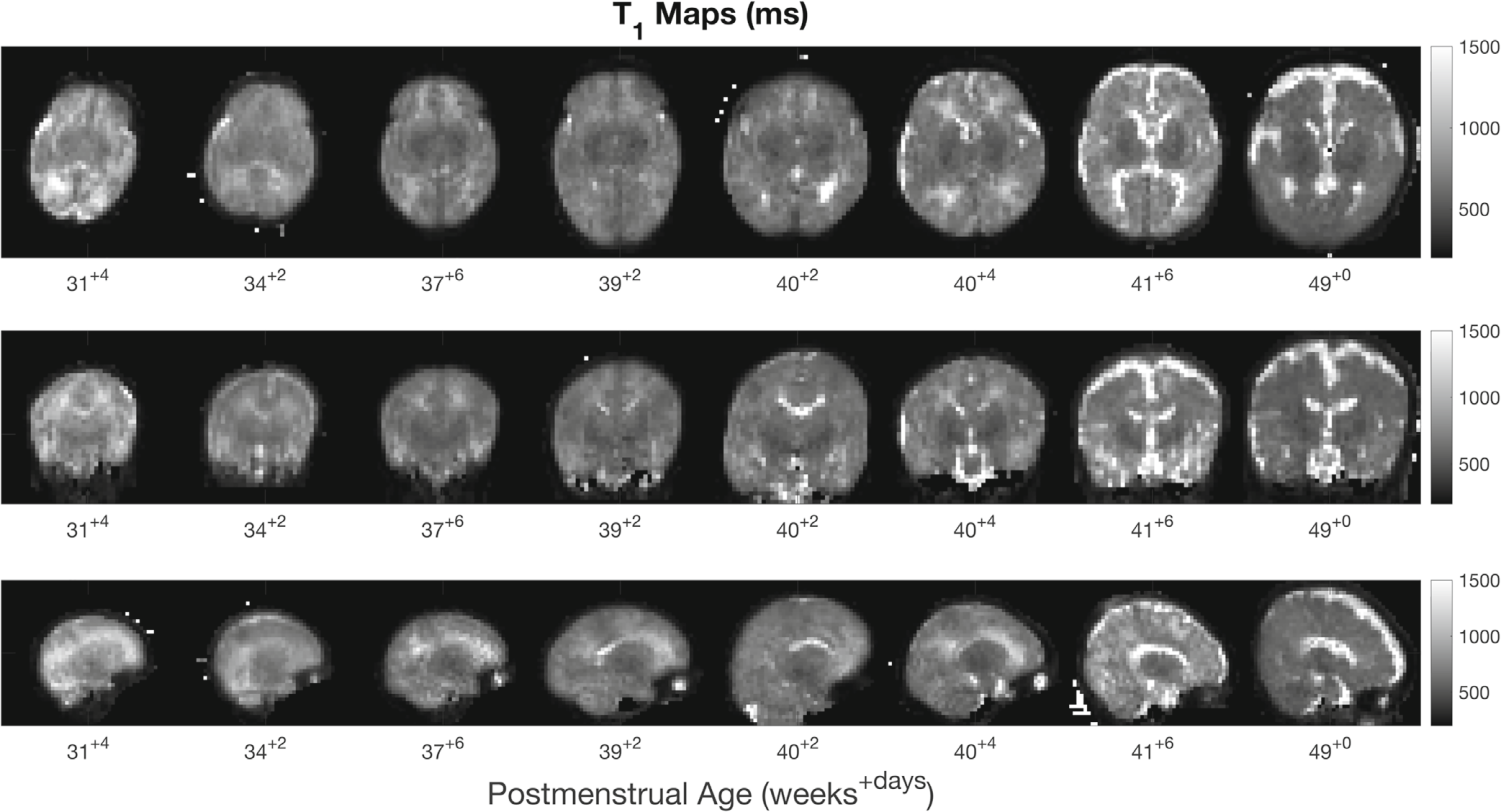
T_1_ maps of 8 subjects over the full age range of neonates scanned in this study

Example T_1_ maps for 8 subjects ordered by postmenstrual age at scan in weeks 31–49 are shown in Figure 6. Maps have isotropic voxel size and demonstrate the ability to differentiate white matter, gray matter, and CSF spaces.

**FIGURE 7:**
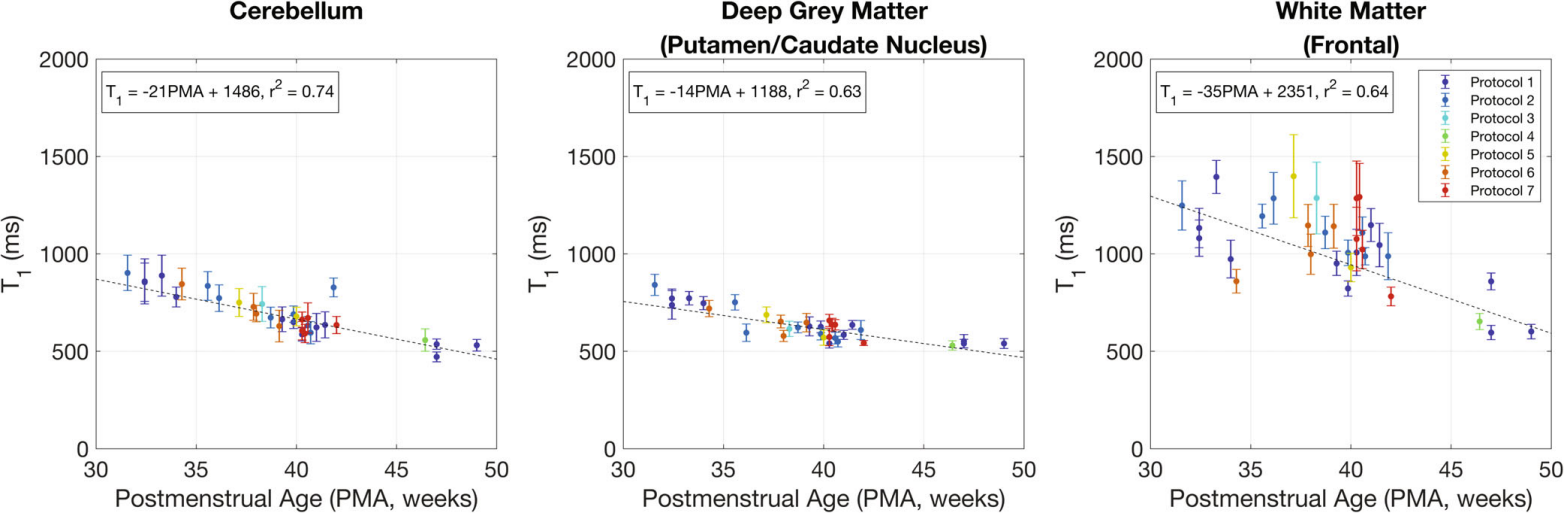
T_1_ versus postmenstrual age. The color of each datapoint indicates the acquisition protocol from Table 1

The T_1_ values plotted against postmenstrual age are shown in Figure 7. Reduction of T_1_ with postmenstrual age is observed in all three brain regions, with the change in T_1_ per week and 95% confidence intervals given by dT_1_ = −21 ms/week [−25, −16] (cerebellum), dT_1_ = −14 ms/week [−18, −10] (deep gray matter), and dT_1_ = −35 ms/week [−45, −25] (white matter).

## 4 DISCUSSION

This study presents the first results from an acquisition method and novel reconstruction pipeline to achieve motion‐compensated T_1_ relaxation rate mapping in the neonatal brain at 64 mT.

The proposed methodology used an inversion‐recovery turbo spin echo for image acquisition. This sequence was chosen due to its widespread use in prior T_1_ mapping publications, including at ULF.^26^ While individual sequence acquisition times were short (3 min 32 s or 7 min 4 s, depending on TR), the overall protocols ranged from 21 to 32 min. Although this extended protocol duration is viable for a research study, future work could use methods that acquire data in a shorter amount of time, allowing for clinical use when time is more limited.^27^

The present study used a varying protocol across our cohort. This reflected the practicalities of imaging neonates in a neonatal unit where a scan may be terminated early to prioritize other clinical work, as well as intentional modifications to the protocol to improve accuracy for longer relaxation times. Future work will use a protocol that is optimized using the appropriate efficiency metric^28^ for the range of T_1_s we have observed in neonates, accounting for a desired protocol duration as well as the possibility of individual sequence failures due to motion, and the fact that any protocol may be truncated due to competing clinical pressures.

The results show that measured neonatal brain T_1_ values at ULF are shorter than those at standard clinical field strengths,^12^, ^13^, ^14^, ^29^ but longer than those of adults at ULF.^26^ The results also show that T_1_ reduces with postmenstrual age, which is thought to reflect an interplay between maturation and key facets of brain‐tissue composition including water content and myelination. Although this trend is consistent with literature obtained at higher fields,^13^, ^29^ at ULF we observe larger fractional reductions in T_1_ than at higher field. Schneider et al^13^ found that normative T_1_ values at 3 T in the thalamus and frontal white matter dropped 11% and 4%, respectively, between approximately 30 and 40 weeks′ gestation, whereas at 64 mT the measured T_1_ reduces in the deep gray matter and frontal white matter by 18% and 26%, respectively.

While this study performed T_1_ mapping in vivo, the paper by Koenig^18^ presented values from unfixed samples excised from a 4‐day‐old neonate who had died due to meconium aspiration. Ex vivo samples from the cortical gray matter and subcortical white matter of the subject′s left parietal lobe had T_1_ values at 64 mT of 494 ms and 655 ms, respectively. Although the gray‐matter sample is consistent with our results (529–841 ms), the white‐matter sample (taken 4 days after delivery) is considerably lower than our measurements at that age range (781–1292 ms).

Future work will use the presented methodology to explore T_1_ variation in more extremely premature infants, in addition to investigating the impact of mode of delivery, age of infant from birth, and pathology. Furthermore, we hope to use these methods to optimize T_1_‐weighted structural imaging in this population, as well as to validate measures of brain development that can be used wherever ULF point‐of‐care MRI can be deployed.

## FUNDING INFORMATION

The Bill and Melinda Gates Foundation; the Medical Research Council (MRC; MR/V036874/1); the Wellcome/EPSRC Center for Medical Engineering (WT 203148/Z/16/Z); a Medical Research Council Center for Neurodevelopmental Disorders grant (MR/N026063/1); an MRC Clinician Scientist Fellowship (MR/P008712/1); an MRC Translation Support Award (MR/V036874/1); and a Sir Henry Dale Fellowship jointly funded by the Wellcome Trust and the Royal Society (206675/Z/17/Z)

## CONFLICT OF INTEREST

Francesco Padormo was employed by the Guy′s & St. Thomas′ NHS Foundation Trust during experimental design, recruitment, all infant data collection, and before submission of the manuscript, but is now an employee of Hyperfine Inc. Lori Arlinghaus, John Pitts, Tianrui Luo, and Dingtian Zhang are employed by Hyperfine Inc.

## Supporting information


**Video S1** Top row: Coronal images before motion compensation. Contrast changes across the volume are seen due to varying sequence parameters, and interimage motion is observed. Bottom row: After intervolume motion compensation, the brain remains static in the frame. Nonlinear motion is still observed outside of the head but is not relevant for T_1_ mapping in the brain

**Video S2** Top row: Sagittal images before motion compensation. Contrast changes across the volume are seen due to varying sequence parameters, and interimage motion is observed. Bottom row: After intervolume motion compensation, the brain remains static in the frame. Nonlinear motion is still observed outside of the head, but is not relevant for T_1_ mapping in the brain
