# Supplementary figures and images for: In vivo T1 mapping of neonatal brain tissue at 64 mT
Source: Magn Reson Med. 2022 Nov 13;89(3):1016–25. doi: 10.1002/mrm.29509 (PMC10099617; doi:10.1002/mrm.29509)

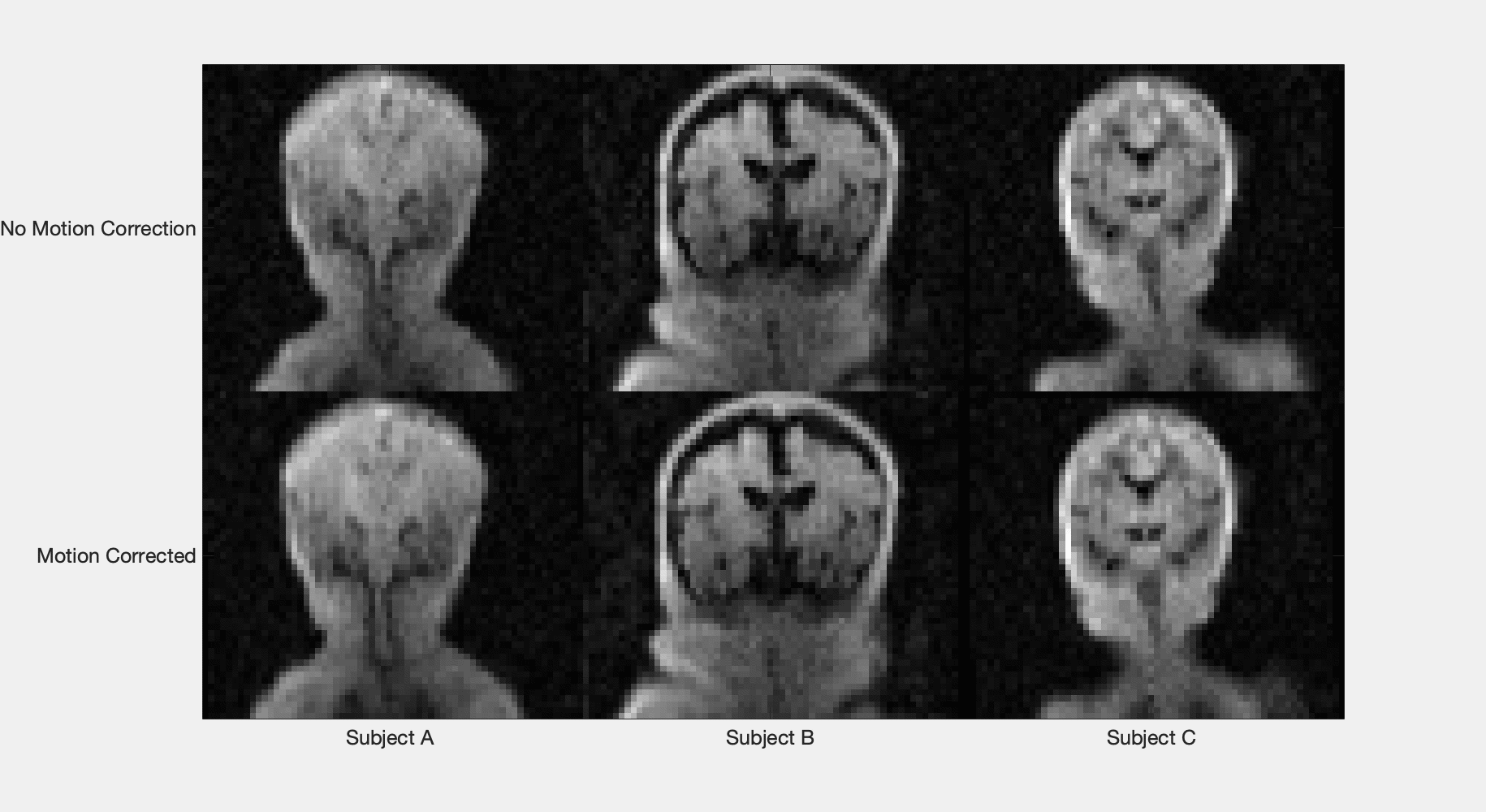

Supplement: Supplementary file 1 — Video S1 Top row: Coronal images before motion compensation. Contrast changes across the volume are seen due to varying sequence parameters, and interimage motion is observed. Bottom row: After intervolume motion compensation, the brain remains static in the frame. Nonlinear motion is still observed outside of the head but is not relevant for T1 mapping in the brain [file MRM-89-1016-s001.gif]

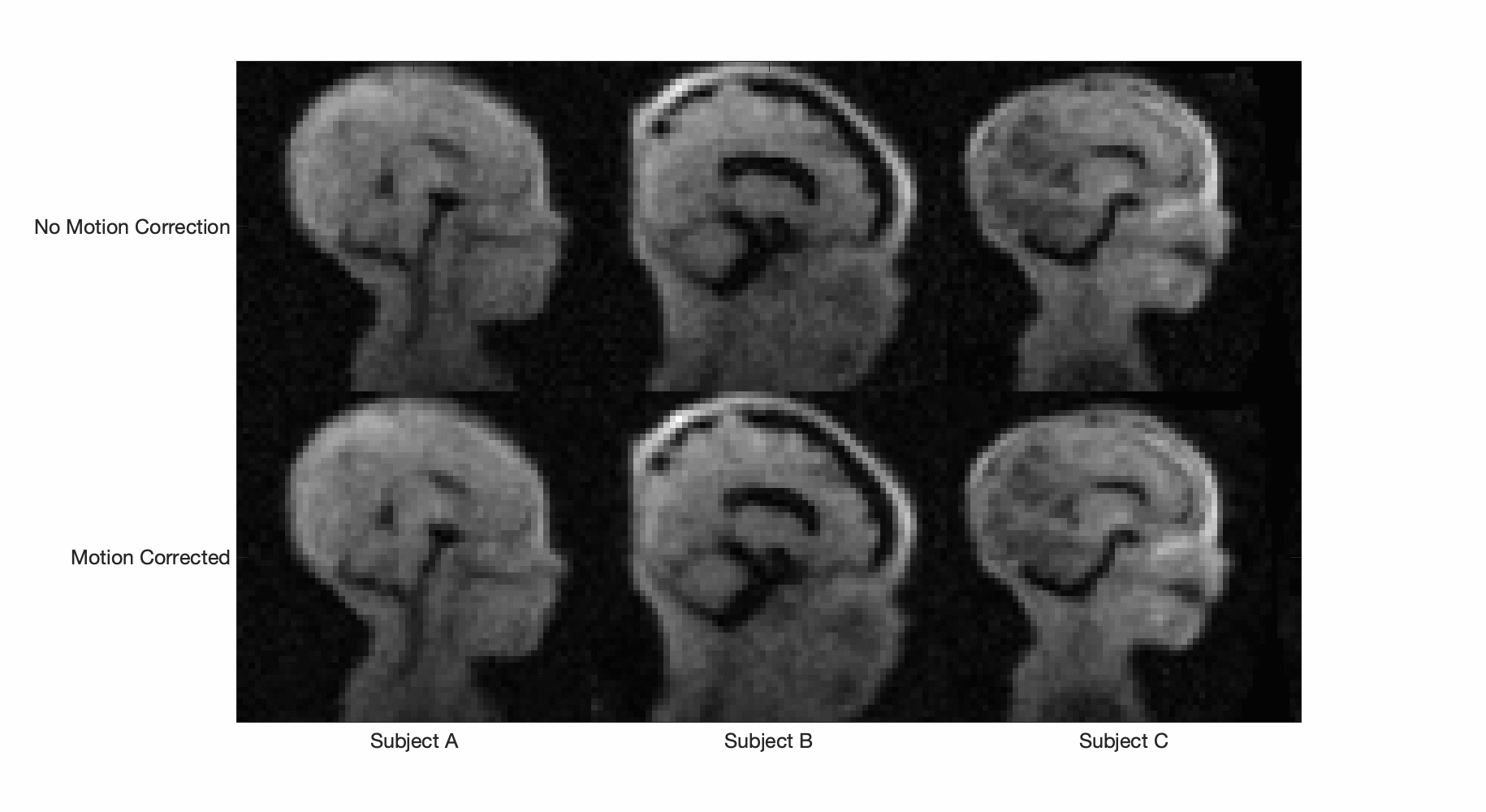

Supplement: Supplementary file 2 — Video S2 Top row: Sagittal images before motion compensation. Contrast changes across the volume are seen due to varying sequence parameters, and interimage motion is observed. Bottom row: After intervolume motion compensation, the brain remains static in the frame. Nonlinear motion is still observed outside of the head, but is not relevant for T1 mapping in the brain [file MRM-89-1016-s002.gif]
